# A case of canine renal lymphoma of granular lymphocytes with severe polycythemia

**DOI:** 10.1186/s12917-021-02854-5

**Published:** 2021-04-14

**Authors:** Sara Kotb, Carolina Allende, T. William O’Neill, Krista Bruckner, Helio DeMorais, Jana Gordon, Kaitlin Curran, Duncan S. Russell, Susanne M. Stieger-Vanegas, Jennifer L. Johns

**Affiliations:** Carlson College of Veterinary Medicine, Oregon State University, Corvallis, OR USA

## Abstract

**Background:**

Renal lymphoma in dogs is rare and has a poor prognosis. Granular lymphocyte morphology is rarely reported in canine renal lymphoma. Mild to moderate polycythemia is reported in a number of canine renal lymphoma cases.

**Case presentation:**

A 10-year-old Labrador retriever presented to a university veterinary teaching hospital after a 1-month history of polyuria, polydipsia, and pollakiuria and a 2-week history of abdominal distention, lethargy, and increased respiratory effort. Abdominal ultrasound showed a wedge-shaped to rounded, heterogeneously hypoechoic mass lesion in the left kidney. Cytologic analysis of a percutaneous aspirate of the mass was consistent with lymphoma of granular lymphocytes. Severe polycythemia (hematocrit 0.871) was noted on a complete blood cell count. Clonality analysis identified a clonally rearranged T-cell receptor (TCR) gene and immunohistochemical staining was CD3+, CD79a- and CD11d+, supporting cytotoxic T-cell lymphoma.

**Conclusions:**

To our knowledge, this is the first report of renal cytotoxic T-cell lymphoma with severe polycythemia in a dog. Severe polycythemia and renal cytotoxic T-cell lymphoma are both rare in dogs; this report adds to the body of knowledge on these conditions.

## Background

Renal lymphoma in dogs is rare and has a poor prognosis [1, 2]. Granular lymphocyte morphology is also unusual in canine primary renal lymphoma; in one report, one out of 29 cases of primary renal lymphoma in dogs had granular lymphocyte morphology indicating a cytotoxic phenotype [2]. Polycythemia is occasionally reported in canine renal lymphoma and is usually mild to moderate in severity. This report presents an unusual case of renal T-cell lymphoma of large granular lymphocytes with concurrent severe polycythemia in a dog.

## Case presentation

A 10-year-old, 30 kg, spayed female Labrador retriever presented to the Small Animal Internal Medicine Service at Oregon State University’s Veterinary Teaching Hospital (OSU VTH) for reported abdominal distension of approximately 2 weeks’ duration.

The dog initially presented to the referring veterinarian for 1 month of polydipsia, polyuria, and pollakiuria. Proteinuria was found on urinalysis and the dog was placed on a two-week course of amoxicillin for presumptive urinary tract infection. The dog continued to exhibit signs of polydipsia, polyuria, and pollakiuria after the medication course was completed. Additionally, she developed visible abdominal distension and increased respiratory effort over 2 weeks. The dog was not on any medications at the time of presentation to OSU VTH.

On physical examination at OSU VTH, the abdomen was tense with signs of pain on palpation. Moderate splenomegaly was noted on palpation. No overt fluid wave was detected. The dog’s vital signs were within the normal range. The peripheral lymph nodes were normal in size on palpation.

Complete blood cell count revealed severe polycythemia (hematocrit = 0.871, reference interval 0.37–0.55; total red blood cell count 13.39 × 10^12^/L, reference interval 5.5–8.5 × 10^12^/L; hemoglobin 17.0 mmol/L, reference interval 7.45–11.17 mmol/L). Marked lymphopenia (0.235 × 10^9^/L, reference interval 1.000–4.800 × 10^9^/L), neutrophil hyposegmentation without increased band neutrophils, and moderate neutrophil toxic changes were noted. Serum biochemistry results showed a mild increase in creatine kinase at 551 U/L (reference interval 50–300 U/L) with other parameters within normal limits. Urine was collected by cystocentesis and showed isosthenuria (specific gravity of 1.010) and proteinuria (3+ protein). Urine sediment examination was unremarkable. Bacterial culture of the urine showed no growth.

**Fig. 1:**
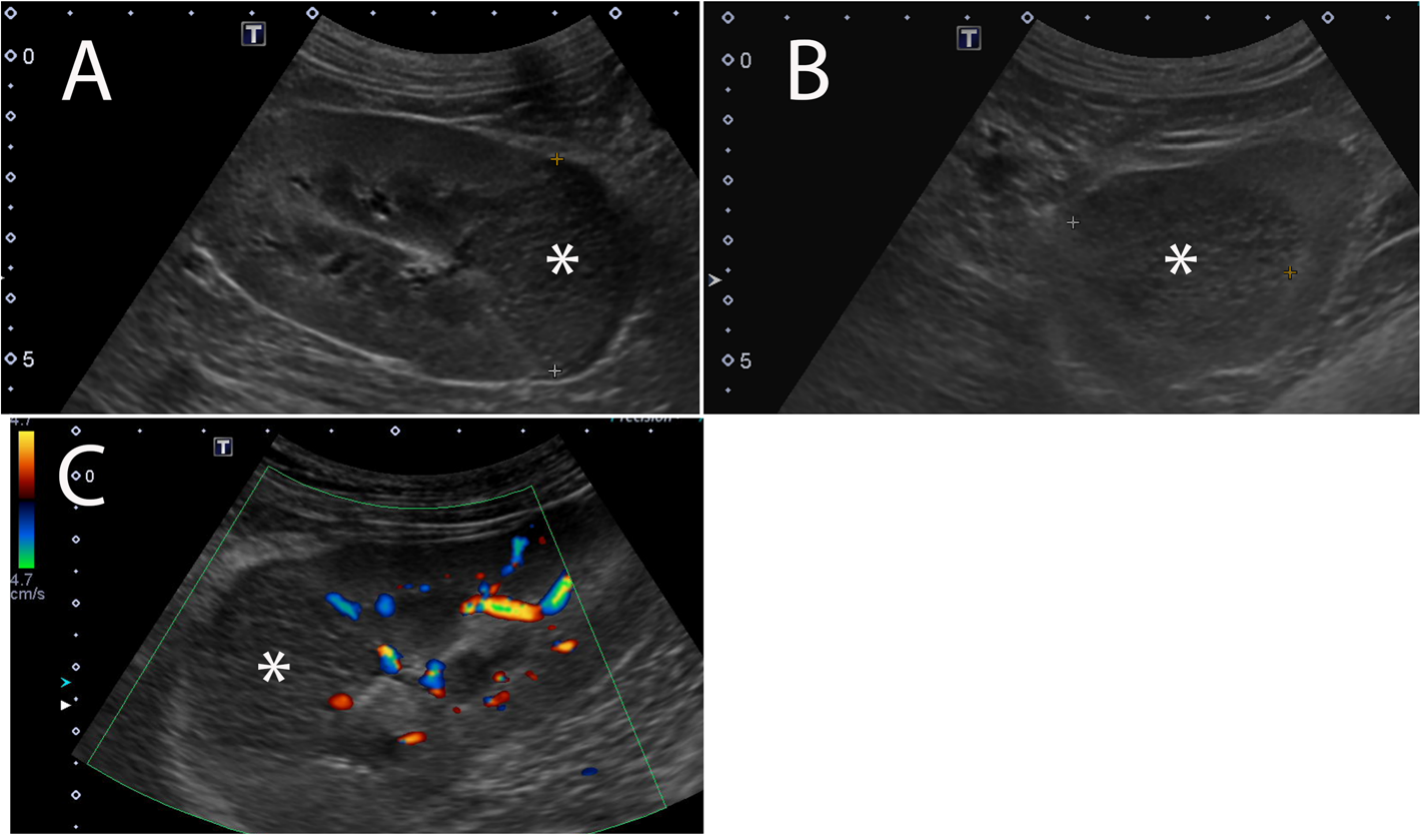
Ultrasound images of left kidney. Transverse (**a**) and sagittal (**b**) B-mode and sagittal color Doppler (**c**) ultrasound images of the left kidney. **a-b**: a round to wedge-shaped, peripherally broad-based, heterogeneously hypoechoic region (white asterisk) from the mid-aspect to caudal pole of the left kidney distorts normal shape of the kidney. **c**. In this hypoechoic region, a reduced color Doppler signal suggesting reduced vascular flow was noted

On abdominal ultrasound, extending from the mid to caudal aspect of the left kidney, there was a medium sized (3.5 × 3.2 cm), round to wedge-shaped, peripherally broad-based, heterogeneously hypoechoic region that caused mild bulging of the caudal margin of the kidney (Fig. 1a-b). Within the wedge-shaped region, there was reduced color Doppler signal (Fig. 1c). Additionally, along the caudal peripheral margin of the kidney, a thin hypoechoic rim was present. The adjacent retroperitoneal fat was mildly hyperechoic. The cranial aspects of both kidneys were normal in margination and echogenicity. The caudal aspect of the right kidney was small and lobular in margination with several, well-defined, concave defects. Bilaterally, renal pelvic enlargement was not identified. No free peritoneal fluid or gas was noted. The liver was subjectively mildly enlarged and diffusely moderately heterogeneous with multifocal hypoechoic nodules ranging from 0.3 to 1.0 cm in diameter. The abdominal lymph nodes including the sublumbar lymph nodes were sonographically normal and normal in size with uniform echogenicity. The spleen was sonographically normal and had uniform echogenicity, normal size and well defined capsular margins. The left renal mass was attributed to either neoplasia (e.g., carcinoma, lymphoma), or acute or recent infarction. The right renal cortical irregularity and reduced size were consistent with renal scarring or previous infarction. Primary diagnostic considerations for the hepatic nodules were nodular hyperplasia and malignant neoplasia (e.g., metastatic or multicentric neoplasia).

**Fig. 2:**
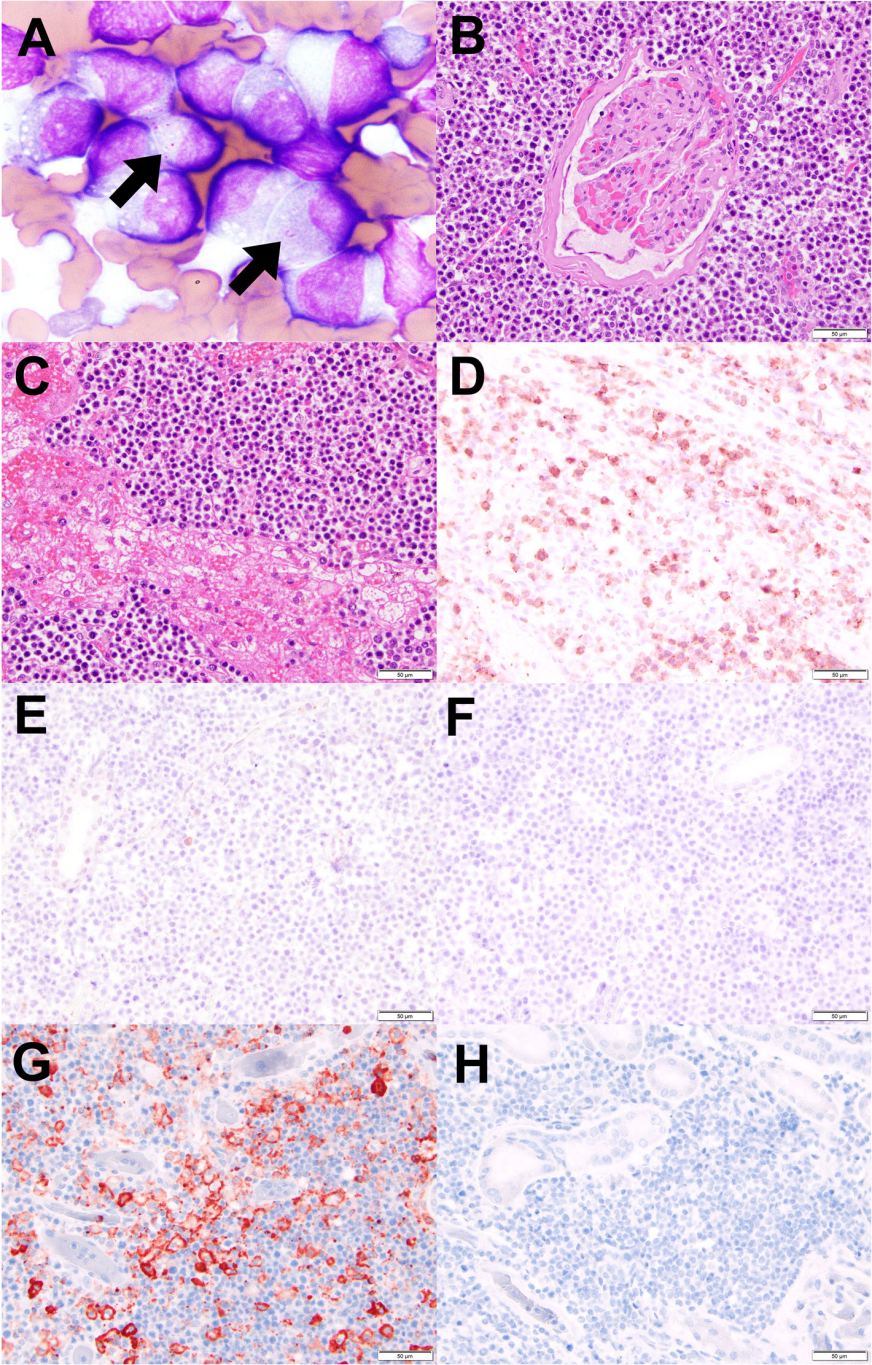
Cytology, histology and immunohistochemistry of left kidney and histology of liver. **a** Magnified cytologic image taken at 100X magnification of fine needle aspirate smears from left kidney (arrows indicate fine granules within neoplastic lymphocytes); B-H are histologic images taken at 400x with scale bar = 50 μm; **b-c** H&E stained section of (**b**) left kidney and (**c**) liver demonstrating neoplastic round cell infiltration; **d-h** immunohistochemical staining (IHC) of left kidney sections: **d** IHC for CD3 demonstrating strong positivity; **e** IHC for CD79a demonstrating negative staining of the neoplastic population; **f** negative control for CD3; **g** IHC for CD11d demonstrating strong positivity; **h** negative control for CD11d

Fine-needle aspiration of the left renal mass found a homogeneous population of intermediate to large-sized lymphocytes (approximately 2 to 2.5 times the diameter of a small mature lymphocyte) with round to clefted nuclei, finely stippled chromatin and indistinct nucleoli (Fig. 2a). 25–50% of cells contained few fine, packeted bright pink intracytoplasmic granules (Fig. 2a, arrows). Renal epithelial cells were also noted. The cytological features were consistent with lymphoma, and lymphoma of granular (cytotoxic) lymphocytes was considered probable. PCR for antigen receptor rearrangements (PARR) was performed on renal cytology smears by Colorado State University’s Veterinary Diagnostic Lab and indicated a clonally rearranged T-cell receptor gene. The combination of cytologic findings and clonality testing results confirmed cytotoxic T-cell lymphoma. Aspiration of a popliteal lymph node was performed to rule out peripheral node involvement in the absence of palpable enlargement, and the cytologic interpretation was consistent with mild reactivity.

Therapeutic phlebotomy was performed to reduce the hematocrit value. 450 ml of blood was removed and 900 ml of Lactated Ringer’s solution infused intravenously. Hematocrit post-phlebotomy decreased to 0.73. The owner declined further staging and chemotherapy, and opted for palliative treatment. Prednisone was prescribed at a dose of 20 mg per os (0.65 mg/kg) once daily.

The dog presented for a follow-up appointment 3 weeks later. On examination, the abdomen was tense, moderately distended and painful. Marked splenomegaly was noted on palpation and attributed to increased erythropoiesis. A complete blood count showed persistent marked polycythemia (hematocrit 0.78). The owner reported improvement in the dog’s attitude and energy since phlebotomy and initiation of glucocorticoid treatment. The owner again declined chemotherapy including hydroxyurea and opted to continue glucocorticoid therapy. Approximately 6 weeks after the dog’s initial presentation to OSU VTH, the owner reported worsening signs of lethargy and elected humane euthanasia. The owner consented to a necropsy.

At necropsy, gross examination confirmed a round to wedge-shaped mass at the caudal pole partially effacing the left kidney. Gross appearance was compatible with the ante-mortem diagnosis of renal lymphoma. In the right kidney, multifocal chronic renal infarcts with fibrosis were noted grossly. In the lungs, scattered 1–2 mm diameter, hard nodules were randomly distributed throughout and were interpreted as consistent with osseous metaplasia. No other gross lesions or abnormalities were found on complete necropsy. Histopathologically, the mass in the left kidney consisted of sheets of neoplastic lymphocytes (Fig. 2b). The cells had a high nucleus to cytoplasm ratio, a large nucleus (approximately 3 times the diameter of an erythrocyte), and significant anisokaryosis. Mitotic figures were infrequent. Histopathology of the liver showed multifocal to coalescing sheets of neoplastic lymphocytes that disrupted hepatic architecture (Fig. 2c) and were similar to those in the left kidney. Histopathologic interpretation was lymphoma with extensive left renal and hepatic involvement. Glycogenosis was also noted in the liver. Histopathologic evaluation of the spleen found three nodules of clustered, hemosiderin-laden macrophages along the splenic capsule (hemosiderotic plaques) associated with a small amount of capsular hemorrhage; and a moderate amount of extramedullary hematopoiesis. Splenic lymphoid architecture was preserved and unremarkable. The remaining histopathologic lesions were diffuse congestion and occasional intravascular fibrin thrombi in the lung, adrenal vasculature congestion, and thin gastric mucosa with scattered spirochete bacteria along the surface (consistent with *Helicobacter* spp.). All other tissues evaluated (brainstem, midbrain, cerebellum, cerebrum, thyroid, colon, and heart) were unremarkable. Histologic sections of the left kidney were immunohistochemically stained for expression of CD3 (Agilent Technologies, Inc., Santa Clara, CA) and CD79a (Novus Biologicals, Centennial, CO) by Oregon Veterinary Diagnostic Laboratory at Oregon State University, and CD11d (Leukocyte Antigen Biology Lab, University of California, Davis, Davis, CA) by University of Minnesota Veterinary Diagnostic Laboratory. The neoplastic population was strongly positive for CD3 (Fig. 2d) and negative for CD79a (Fig. 2e) with confirmed negative control (Fig. 2f). The neoplastic population was strongly positive for CD11d (Fig. 2g) with confirmed negative control (Fig. 2h).

## Discussion and conclusions

Primary renal neoplasia in the dog is rare with reports accounting for 0.2–1.7% of all identified canine tumors. Renal lymphoma in dogs is most commonly reported bilaterally and rarely unilaterally [2, 3]. Canine primary renal tumors are predominantly of epithelial or embryonal origin; mesenchymal neoplasms account for 11% of tumors and primary renal lymphoma is rarely reported [3, 4]. Canine renal lymphoma is most commonly associated with multicentric lymphoma, making primary lymphoma difficult to differentiate from multicentric with renal involvement [2–6]. In human patients, multiple criteria including histopathology with no other extranodal or nodal sites of involvement are utilized to determine primary renal lymphoma [6]. In veterinary medicine, no clear guidelines have been established and involvement of other extranodal tissues is often accepted [6]. In the current case, the sonographically noted liver changes were nonspecific and differentials considered included benign (e.g. vacuolar hepatopathy, nodular, hyperplasia) or malignant (e.g. metastatic or multicentric neoplasia) etiologies. Hepatic involvement with lymphoma was found at necropsy. Hepatomegaly was noted on ultrasound and splenomegaly was noted on initial and repeat palpation. Both ultrasound and palpation are considered subjective measures of organomegaly; this likely explains the discrepancy between these findings. Quantitative measures of organomegaly (e.g., computed tomography; organ weights on postmortem evaluation) were not performed in this case. Abdominal distention was not noted on initial examination at our hospital and the abdomen was tense and painful. On recheck examination, the abdomen did appear distended. The finding of moderate extramedullary hematopoiesis on post-mortem histopathologic evaluation likely explains the palpated splenomegaly; decreased abdominal muscle tone particularly when the patient was relaxed may have contributed to the owners’ perception of abdominal distention.

Based on imaging and necropsy findings (particularly the large size and unilateral nature of the left kidney mass), primary renal lymphoma with secondary hepatic involvement was considered more likely than lymphoma arising in the liver and extending to the kidney. However, as the liver lesions were not evaluated via cytology or histopathology at the time of ultrasound, primary renal lymphoma cannot be confirmed in this case. Common ultrasonographic features in canine renal lymphoma include loss of corticomedullary distinction, renomegaly, renal deformity, and hypoechoic lesions, all of which were present in this case. The source of the neoplastic population in primary renal lymphoma has been speculated to arise from renal capsular lymphatics or from chronic lymphocytic inflammation leading to neoplastic transformation [3, 6]. The prevalence of primary renal lymphoma is unknown in dogs with relatively few cases reported in the literature [2, 6]. Associated clinical signs tend to be nonspecific; dogs generally present systemically ill with decreased appetite, weight loss, and/or abdominal pain [2]. Most primary renal lymphomas have been identified as T-cell based on CD3+ phenotype and PARR, with rare reports of B-cell origin [2, 6, 7]. Prognosis is generally considered poor. Chemotherapy protocols described for canine primary renal lymphoma include COP (cyclophosphamide, vincristine and prednisolone with or without cytarabine on the first day of treatment) or CHOP (cyclophosphamide, vincristine, prednisolone and doxorubicin with or without L-asparaginase at induction) [1, 2, 8]. Reported survival times are generally short with most dogs succumbing to disease ≤47 days following institution of a combination chemotherapy protocol.

Erythrocytosis is reported with several canine renal neoplasms including primary renal lymphoma [1–3, 7, 9]. Degree of reported erythrocytosis is mild to moderate; to the authors’ knowledge, the highest reported hematocrit is 0.76. In the current case, the dog had severe erythrocytosis with a hematocrit of 0.87. True polycythemia (erythrocytosis) is uncommon and categorized as primary or secondary polycythemia. Primary erythrocytosis (polycythemia vera) is a rare entity associated with increased erythropoiesis due to neoplastic transformation of erythroid precursors that proliferate independent of stimulation by erythropoietin [3, 7, 9]. Secondary erythrocytosis is far more common and is subdivided into appropriate or inappropriate responses [9]. Appropriate responses occur with tissue hypoxia and resultant increased erythropoietin signaling, whereas inappropriate responses are commonly due to erythropoietin-stimulating renal neoplasia [9]. The pathophysiological response associated with neoplasia could be increased production of erythropoietin or erythropoietin-like factors by the neoplastic population and/or increased erythropoietin production secondary to local tissue hypoxia related to neoplastic infiltration [1, 9]. In one report, 15 of 29 cases of canine primary renal lymphoma had concurrent erythrocytosis [2]. Cases of canine presumed primary renal lymphoma with secondary erythrocytosis have an almost exclusively T-cell phenotype, consistent with the findings in this case [2, 7]. However, the marked degree of polycythemia found in this case substantially exceeds what is reported in other patients. Immunohistochemical staining of affected tissue for erythropoietin production is rarely performed in veterinary medicine; in one report, two dogs with primary renal lymphoma had positive erythropoietin expression by the neoplastic population [9]. In the case described here, severe erythrocytosis was likely a secondary inappropriate response resulting from neoplastic production of erythropoietin as the dog was not clinically dehydrated and had a normal plasma protein (ruling out relative polycythemia). However, serum erythropoietin measurement and immunohistochemical staining for erythropoietin were not performed to confirm secondary inappropriate erythrocytosis.

Severe erythrocytosis of the magnitude seen in this case is rare in veterinary and human medicine and can have severe consequences. Hyperviscosity syndrome can develop with potential sequelae including neurological disturbances, bleeding diatheses, glomerulonephritis, uveitis, and other thromboembolic events [10–14]. Activation of the coagulation cascade is a known risk with hyperviscosity syndrome due to polycythemia vera in human patients and may be a concern in canines as well [14, 15]. Therapeutic phlebotomy and volume replacement via crystalloids is the recommended treatment for dogs and cats, though not all animals may require fluid replacement [16]. A target hematocrit in the upper end, or just above, the reference interval (e.g., target hematocrit 0.58–0.65) is suggested for small animals without cyanotic heart disease [17]. Myelosuppressive therapy with hydroxyurea is recommended to treat polycythemia vera, and may be considered to treat secondary erythrocytosis if therapeutic phlebotomy is required more frequently than every 4 weeks and/or is difficult due to hyperviscosity [16]. In this case, therapeutic phlebotomy with fluid replacement therapy was utilized along with glucocorticoid palliative therapy for lymphoma. Additional chemotherapeutic therapy, including hydroxyurea treatment, was declined.

An additional unique feature in this case was granular lymphocyte morphology of neoplastic cells. Granular lymphocytes are often larger than normal lymphocytes and comprise less than 10% of the normal canine circulating lymphocyte population [18, 19]. Granular lymphocytes are further subdivided into CD3- NK cells and CD3+ T cells [19, 20]. Though considered a minor subset in circulation, granular lymphocytes represent a large subset of intestinal intraepithelial lymphocytes in most species [21]. Immunophenotyping has identified distinct T-cell subpopulations subdivided by T-cell receptor type: αβ T cells (the large majority of T cells overall) and γδ T cells (< 1–2% in circulation) [20, 22, 23]. The γδ T cells are a post-thymic T cell population that largely localize in the spleen, with lower numbers found in other tissues [19, 23–26]. The function of γδ T cells is believed to be a first line of defense in epidermal and mucosal epithelial lining [23, 25].

In people, ‘large granular lymphocyte’ tumors tend to be aggressive with high mortality, and are often associated with extranodal locations [18]. World Health Organization criteria classify these neoplasms in human patients based on clinical, morphological, immunophenotypic, and genetic criteria [26]. The incidence of ‘large granular lymphocyte’ tumors in dogs is low (< 1.8%) with a similarly aggressive clinical course to that of humans [18, 27]. In dogs, neoplasia of granular lymphocytes often presents as chronic lymphocytic leukemia of variable clinical progression with rare cases affecting single or multiple organs (often liver and spleen), and is thought to often originate in the spleen [19, 20, 22, 26, 28]. Two distinct entities in dogs, hepatosplenic and hepatocytotropic T-cell lymphoma, are described with the majority identified as γδ phenotype; rare renal involvement is noted in one study [18, 22, 26, 29].

To the authors’ knowledge, only rare reports have suggested primary renal lymphoma of granular lymphocytes; minimal information was provided previously regarding immunophenotyping and further characterization [2, 30]. In the current case, CD3 and CD11d positivity and lack of CD79a positivity (ruling out B-cell phenotype) support T-cell lymphoma of granular lymphocytes. CD3 immunoreactivity is established as a marker of T cells in dogs and many other species; in dogs, CD11d immunoreactivity occurs on macrophages and T cells in the splenic red pulp and on large granular lymphocytes [28]. No reports of renal granular lymphocyte lymphoma were found with the degree of erythrocytosis identified in this case. Differentiation of αβ and γδ T-cell receptor expression can be performed via immunophenotyping (flow cytometric analysis or immunocytochemical staining) to further characterize cytotoxic T cell populations, but was not performed in this case.

In summary, this case demonstrates a unique presentation of aggressive canine cytotoxic T-cell lymphoma with renal involvement and severe polycythemia presumed to be secondary inappropriate erythrocytosis.

## Data Availability

The datasets and images used and/or analyzed during the current study are available from the corresponding author on reasonable request.
